# Case report: Therapy-related myeloid neoplasms in three pediatric cases with medulloblastoma

**DOI:** 10.3389/fonc.2024.1364199

**Published:** 2024-03-26

**Authors:** Li Shun Mak, Xiuling Li, Wilson Y. K. Chan, Alex W. K. Leung, Daniel K. L. Cheuk, Liz Y. P. Yuen, Jason C. C. So, Shau Yin Ha, Anthony P. Y. Liu

**Affiliations:** ^1^ Department of Paediatrics and Adolescent Medicine, Princess Margaret Hospital, Hong Kong, Hong Kong SAR, China; ^2^ Department of Pediatrics and Adolescent Medicine, Hong Kong Children’s Hospital, Hong Kong, Hong Kong SAR, China; ^3^ Department of Pathology, Hong Kong Children’s Hospital, Hong Kong, Hong Kong SAR, China; ^4^ Department of Paediatrics and Adolescent Medicine, School of Clinical Medicine, Li Ka Shing Faculty of Medicine, The University of Hong Kong, Hong Kong, Hong Kong SAR, China

**Keywords:** case report, medulloblastoma (MB), therapy-related myelodysplastic syndrome/acute myeloid leukemia, chemotherapy, alkylating agent, genetic predisposition, Li-Fraumeni syndrome

## Abstract

**Introduction:**

Medulloblastoma is the most common malignant brain tumor in children, often requiring intensive multimodal therapy, including chemotherapy with alkylating agents. However, therapy-related complications, such as therapy-related myeloid neoplasms (t-MNs), can arise, particularly in patients with genetic predisposition syndromes. This case report presents three pediatric cases of medulloblastoma with subsequent development of t-MNs, highlighting the potential role of genetic predisposition and the importance of surveillance for hematological abnormalities in long-term survivors.

**Case presentation:**

We describe three cases of pediatric medulloblastoma who developed t-MNs after receiving chemotherapy, including alkylating agents. Two of the patients had underlying genetic predisposition syndromes (*TP53* pathologic variants). The latency period between initial diagnosis of medulloblastoma and the development of secondary cancer varied among the cases, ranging from 17 to 65 months. The three cases eventually succumbed from secondary malignancy, therapy-related complications and progression of primary disease, respectively.

**Conclusions:**

This report highlights the potential association between genetic predisposition syndromes and the development of therapy-related myeloid neoplasms in pediatric medulloblastoma survivors. It underscores the importance of surveillance for hematological abnormalities among such patients.

## 1 Introduction

Medulloblastoma is a common brain tumor among children (1). The standard treatment includes surgical resection and chemoradiotherapy. However, chemotherapy drugs used in its treatment, particularly alkylating agents and topoisomerase II inhibitors, have been linked to the development of secondary hematological malignancies (2, 3). Additionally, certain genetic predisposition syndromes, such as Li Fraumeni Syndrome (LFS), may increase the risk of multiple primary tumors (4, 5), and therapy-related malignancies. We present (**Table 1**) three children who first suffered from medulloblastoma and developed myeloid neoplasms shortly after treatment completion of the brain tumor.

**Table 1:** Summary of clinical histories.

	Medulloblastoma	Germline studies	Age at Dx	Treatment	2nd cancer	Treatment	3rd cancer	Treatment	Outcome, time after MB, age
1	Desmoplastic/nodular MB (standard risk) Pathology: Non-amplified *MCYN* *TP53* wildtype SHH-MB	*De novo TP53* pathologic variant from buccal swab Parents tested	8y	Packer’s protocol (Lomustine, vincristine, cisplatin) CSI (23.4Gy) + tumor bed boost (30.6Gy) Total 54 Gys	Myelodysplastic neoplasm with biallelic TP53 inactivation, germline TP53 LP variant, post cytotoxic therapy (Dicentric chromosome 5, 17) MB: no evidence of recurrence 17 months from MB	Azacitidine (2 courses) Haploidentical HSCT 4 courses Conditioning regimen: • Alemtuzumab 0.6mg/kg • Fludarabine 150mg/m2 • Thiotepa 10mg/kg • Melphalan 140mg/m2	T-LBL, stage III MB: no evidence of recurrence 45 months from MB	Methotrexate, asparaginase, vincristine, daunorubicin	Died from T-LBL (3^rd^ cancer) 48 months from diagnosis 12y
2	Focal anaplastic features Pathology: Non-amplified *MYCN* *TP53* mutant SHH-MB	*TP53* pathologic variant from EDTA blood Parents not tested	9y	Packer’s protocol (Lomustine, vincristine, cisplatin) Withheld at cycle 7 due to PRES CSI (36Gy cranial + 36Gy spinal)	Myelodysplastic neoplasm with biallelic TP53 inactivation, germline TP53 LP variant, post cytotoxic therapy (5q loss, monosomy 7, trisomy 8, 17p loss) MB: no evidence of recurrence 33 months from MB	Double cord blood transplantation Conditioning regimen: • Cyclophosphamide 120mg/kg • Busulfan dose 12.8 mg/kg • Antithymocyte globulin 5mg/kg • Melphalan 140mg/m^2^			Died from complication of HSCT 43 months from diagnosis 13y
3	Classic medulloblastoma Pathology: Non-amplified *MYCN* Group 3 46 months: Recurrent anaplastic medulloblastoma Variant allele 47.8%, for *MUTYH* protein, unknown significance	*TP53* mutation not reported from EDTA blood Parents not tested	5y	1. HKPHOSG PNET-CNS-2000 protocol Cisplatin, vincristine, lomustine Proton Stereotactic Body Radiation Therapy (23.4Gy) and tumor bed boost (1-1.5cm margin). Total 54 Gys 2. MEMMAT protocol Bevacizumab, thalidomide, celecoxib, fenofibrate (oral) Etoposide, cyclophosphamide (systemic) Etoposide, cytarabine (intraventricular) SBRT of recurrent lesions in left frontal region (54Gy) CAR-T cell therapy anti-GD2	Acute myeloid leukemia, myelodysplasia-related, post cytotoxic therapy (Monosomy 7) MB: disease progression with BM involvement 65 months from MB (18 months from relapse)	Azacitidine, venetoclax (1 course)			Died from complications of medulloblastoma and MDS 69 months from diagnosis 11y

## 2 Case descriptions

### 2.1 Case 1

The first patient was an 8-year-old Chinese boy who underwent surgery for a localized tumor in the cerebellar vermis. Pathology confirmed desmoplastic/nodular medulloblastoma without *MYCN* amplification. The patient received craniospinal irradiation (CSI, 23.4Gy) with a tumor bed boost (30.6Gy) and concurrent vincristine (1.5mg/m^2^ 8 doses), followed by chemotherapy according to Packer’s protocol, which comprised cisplatin (75mg/m^2^), lomustine (75mg/m^2^) and vincristine (1.5mg/m^2^ for 3 doses) for 8 cycles (6). Treatment was generally well-tolerated, with mild hearing loss and hypothyroidism as side effects.

**Figure 1:**
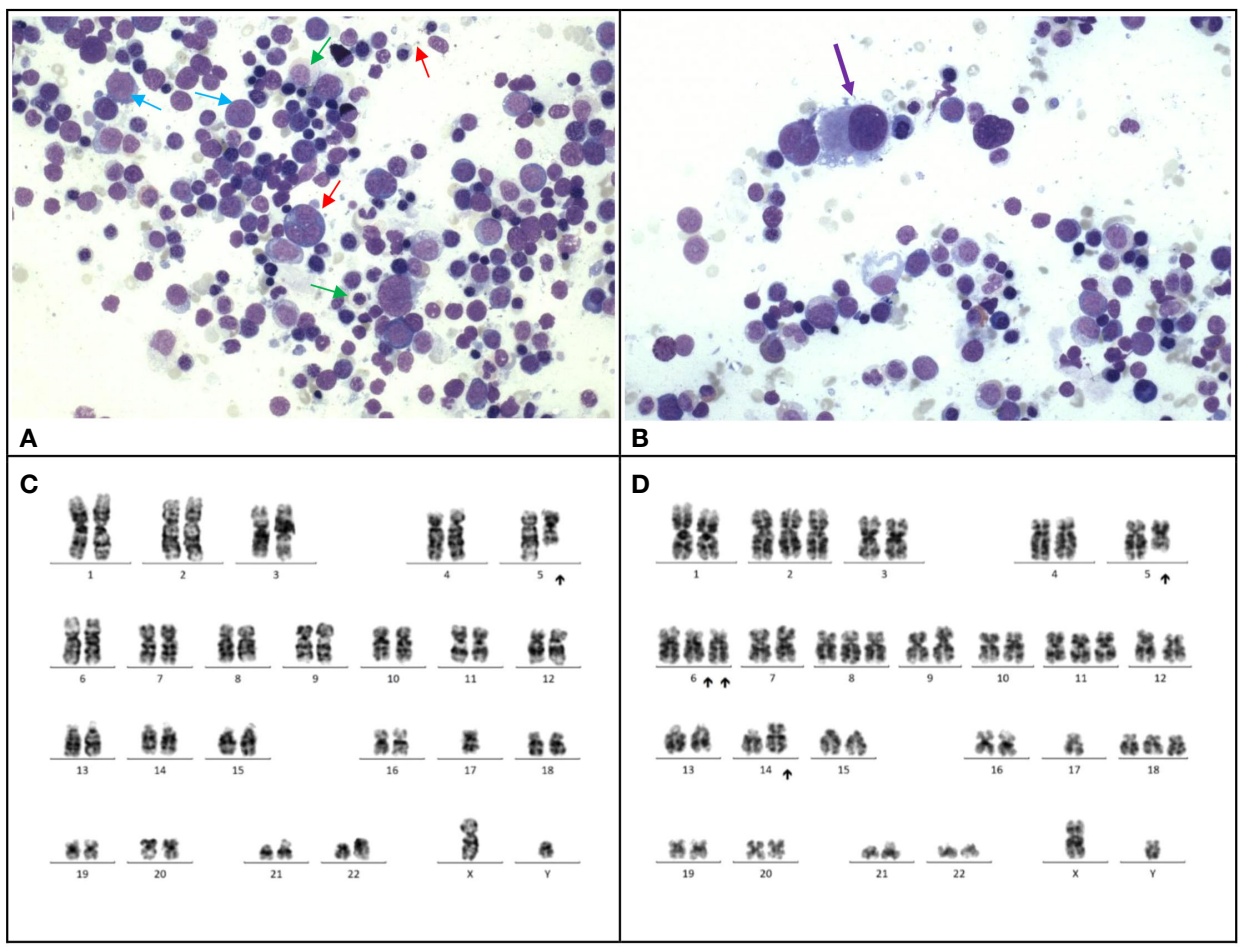
Case 1. **(A)**, The bone marrow demonstrates dyserythropoiesis (red arrows), dysgranulopoiesis (green arrows) and increased blasts (blue arrow). Wright-Giemsa stain, 400 X magnification. **(B)**, The bone marrow shows dysmegakaryopoiesis (purple arrow). Wright-Giemsa stain, 400 X magnification. **(C, D)**, Karyotyping performed on the marrow reveals a complex karyotype with evidence of clonal evolution. **(C)**, The stemline is 45,XY, dic(5;17)(q11.2;p11.2). **(D)**, Clonal evolution to a complex karyotype 50,XY,+2,dic(5;17)(q11.2;p11.2);+6,add(6)(p21)x2,+8,+11,add(14)(p11.2),+18.

At 13 months post-chemotherapy, the patient developed pancytopenia and circulating blasts at 11%. Marrow examination confirmed myelodysplastic neoplasm (with biallelic TP53 inactivation, germline TP53 LP variant, post cytotoxic therapy) (**Figures 1A, B**). **Table 2** Cytogenetic analysis revealed a complex karyotype with dicentric chromosomes 5 and 17 (**Figures 1C, D**). Germline studies from the buccal swab confirmed a *de novo* heterozygous missense likely pathogenic variant in *TP53* (NM_000546.5: c.326T>C, p.Phe109Ser). Genetic investigations for the parents were unremarkable confirming that the *TP53* variant arose *de novo* in the patient. Methylation profiling of the medulloblastoma sample confirmed the Sonic-Hedgehog (SHH) group. The patient received 2 courses of azacytidine, lasting 7 days and 5 days respectively, followed by haploidentical hematopoietic stem cell transplantation with the father as the donor (Conditioning regimen outlined in **Table 1**). After the transplantation, there was no evidence of MDS on bone marrow examination.

**Table 2:** Pathological findings of myeloid neoplasms.

	Diagnosis subtype; Latency	Blasts	Dysplasia findings	Cytogenetics
1	Myelodysplastic neoplasm with biallelic TP53 inactivation, germline TP53 LP variant, post cytotoxic therapy; 13 months from end of chemotherapy	11%	PB: HB 7.4 x 10^12^/L, MCV 69 fL, WBC 3.9 x 10^9^/L, ANC 0.98 x 10^9^/L, PLT 53 x 10^9^/L. Occasional blasts and dysplastic neutrophils. BM: Granulopoiesis shows abnormal nuclear segmentation. Erythropoiesis shows nuclear-cytoplasmic asynchrony, nuclear irregularity, nuclear budding, karyolysis and poor haemoglobinisation. Megakayopoiesis shows some small hypolobulated or hypogranulated forms.	45,XY,dic(5;17)(q11.2;p11.2)[4]/50,idem,+2,+6,add(6)(p21)x2,+8,+11,add(14)(p11.2),+18[11]/46,XY[3]
2	Myelodysplastic neoplasm with biallelic TP53 inactivation, germline TP53 LP variant, post cytotoxic therapy; 19 months from end of chemotherapy	Not increased	PB: HB 9.0 x 10^12^/L, MCV 90 fL, WBC 5.4 x 10^9^/L, ANC 2.9 x 10^9^/L, PLT 71 x 10^9^/L. BM: Markedly hypercellular with trilineage hyperplasia. No dysplasia.	45,XX,del(5)(q31),der(14;17)(q10;q10)[3]/45,XX,der(5;17)(p10;q10),-7,+8[6]/46,XX[15]
3	Acute myeloid leukaemia, myelodysplasia-related, post cytotoxic therapy; 3 months from end of chemotherapy	>20%	PB: HB 7.9 x 10^12^/L, MCV 82 fL, WBC 0.7 x 10^9^/L, ANC 0.36 x 10^9^/L, PLT 40 x 10^9^/L. 4% blasts. Occasional hypogranular neutrophils. BM: Dysgranulopoiesis and dysmegakaryopoiesis; Markedly reduced erythropoiesis	45,XY,-7[27]/46, XY[2]

At 1 year 3 months post-hematopoietic stem cell transplantation (HSCT), the patient developed an anterior mediastinal mass, which was diagnosed as T-lymphoblastic lymphoma. The tumor sample showed a frameshift deletion in exon 34 encoding the PEST domain of the *NOTCH* gene (NM_017617.5:c.7386del p.(Ala2463Profs*14)), in addition to the known germline *TP53* variant. He underwent chemotherapy with vincristine, daunorubicin, methotrexate, and asparaginase, which resulted in hepatorenal toxicities and acute pancreatitis, and only transient tumoral response. The patient passed away 2 months from the diagnosis of lymphoma and 4 years from the initial medulloblastoma diagnosis, due progression of mediastinal mass and subsequent respiratory failure with pleural effusion.

### 2.2 Case 2

The second patient was a 9-year-old Chinese girl diagnosed with a posterior fossa tumor in the superior vermis, extending into bilateral cerebellar hemispheres, with leptomeningeal enhancement and T4 drop metastasis. The patient underwent gross-total resection, and pathology confirmed medulloblastoma with focal anaplasia. *TP53* immunohistochemistry indicated a mutant pattern, while *MYCN* amplification was not detected by fluorescence *in situ* hybridization (FISH). Methylation profiling of the medulloblastoma also revealed Sonic-Hedgehog (SHH) group. The patient received CSI (36Gy cranial + 36Gy spinal) along with concurrent vincristine, followed by chemotherapy according to the Packer’s protocol (6). However, she experienced posterior reversible encephalopathy syndrome after the seventh cycle of chemotherapy, leading to premature termination of treatment.

**Figure 2:**
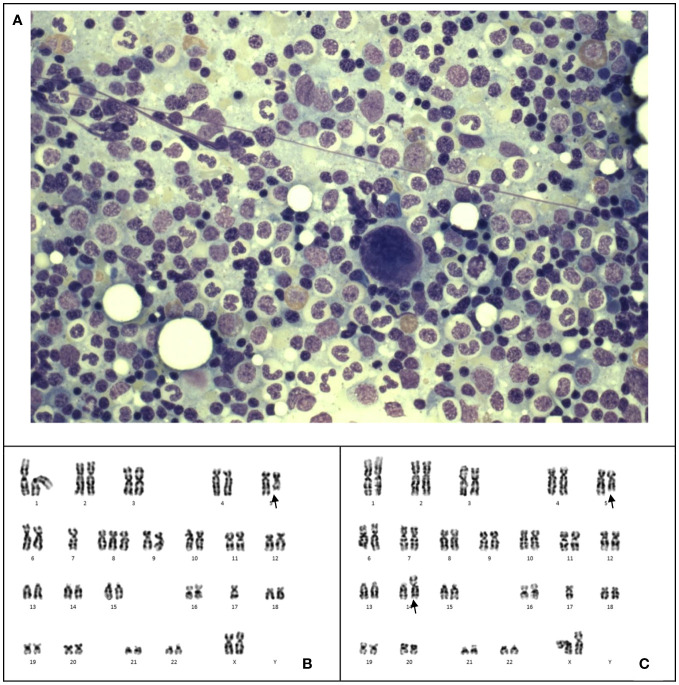
Case 2. **(A)** The bone marrow shows markedly hypercellular marrow with no dysplasia or increase in blasts. Wright-Giemsa stain, 1600 X magnification. **(B, C)**, Karyotyping of the bone marrow reveals two unrelated clones 45,XX,del(5)(q31),der(14;17)(q10;q10)[3]/45,XX,der(5;17)(p10;q10),-7,+8[6]/46,XX[15]. The larger clone **(B)** shows monosomy 7, trisomy 8 and an unbalanced whole arm translocation of 5p and 17q. The latter results in loss of 5q and 17p. The smaller clone **(C)** shows deleted 5q and an unbalanced translocation of 14q and 17q with resultant loss of 17p.

The patient experienced worsening thrombocytopenia and anemia 17 months after end of chemotherapy. The marrow examination revealed myelodysplastic neoplasm (with biallelic TP53 inactivation, germline TP53 LP variant, post cytotoxic therapy) (**Figure 2A**), (**Table 2**) which was evidenced by cytogenetic abnormalities including 5q loss, monosomy 7 and 17p loss (**Figures 2B, C**). Germline study obtained from the peripheral blood showed heterozygous missense likely pathogenic variant in *TP53* (NM_000546.5: c.827C>A, p.Ala276Asp). The patient had a family history of leukemia in a maternal uncle, while parents opted not for germline testing. Methylation profiling confirmed the medulloblastoma molecular group as SHH activated. Double cord blood transplantation was performed (Conditioning regimen in **Table 1**), as near-full donor chimerism of 99-100% had been demonstrated in serial peripheral blood and marrow samples since post-transplant 1 month. The total nucleated cell (TNC) dose was 1.882 x 10^7^/kg and stem cell dose 1.403 x 10^5^ CD34+ cells/kg. Graft-versus-host disease (GVHD) prophylaxis was provided with cyclosporin and mycophenolate mofetil. However, the procedure was complicated by acute graft-versus-host disease (GVHD), adenovirus viremia, and thrombotic microangiopathy. The patient was treated with methylprednisolone, budesonide, infliximab, vedolizumab and extracorporeal photopheresis (ECP) for stage IV acute GVHD. The patient’s condition deteriorated and passed away 10 months after the MDS diagnosis due to fungal pneumonia, 3 years and 7 months since the initial diagnosis of medulloblastoma.

### 2.3 Case 3

The third patient was a 5-year-old Chinese boy diagnosed with a localized cerebellar tumor. He underwent a gross total surgery resection and the pathology identified a group 3 medulloblastoma without MYC amplification. The patient underwent proton stereotactic body radiation therapy (23.4Gy) with tumor bed boost (Total 54Gy) and concurrent vincristine, followed by adjuvant cisplatin, lomustine and vincristine. Treatment lasted for 16 months and resulted in complications including hearing loss, growth hormone deficiency, and hypothyroidism.

46 months from initial diagnosis, metastatic relapse was detected with interval enlargement of a left caudate lesion, further confirmed on biopsy. Germline studies from the peripheral blood revealed a heterozygous variant in *MUTYH* (NM_001048171.1: c.892-2A>G). Genetic analysis of the relapsed tumor showed a variant allele frequency of 47.8% for the same mutation. DNA sequencing of the tumor sample showed no mutation in *TP53*. There was no significant family history of malignancies. The patient received chemotherapy according to the metronomic protocol MEMMAT (etoposide and cyclophosphamide containing) (7) and focal radiotherapy (54Gy).

**Figure 3:**
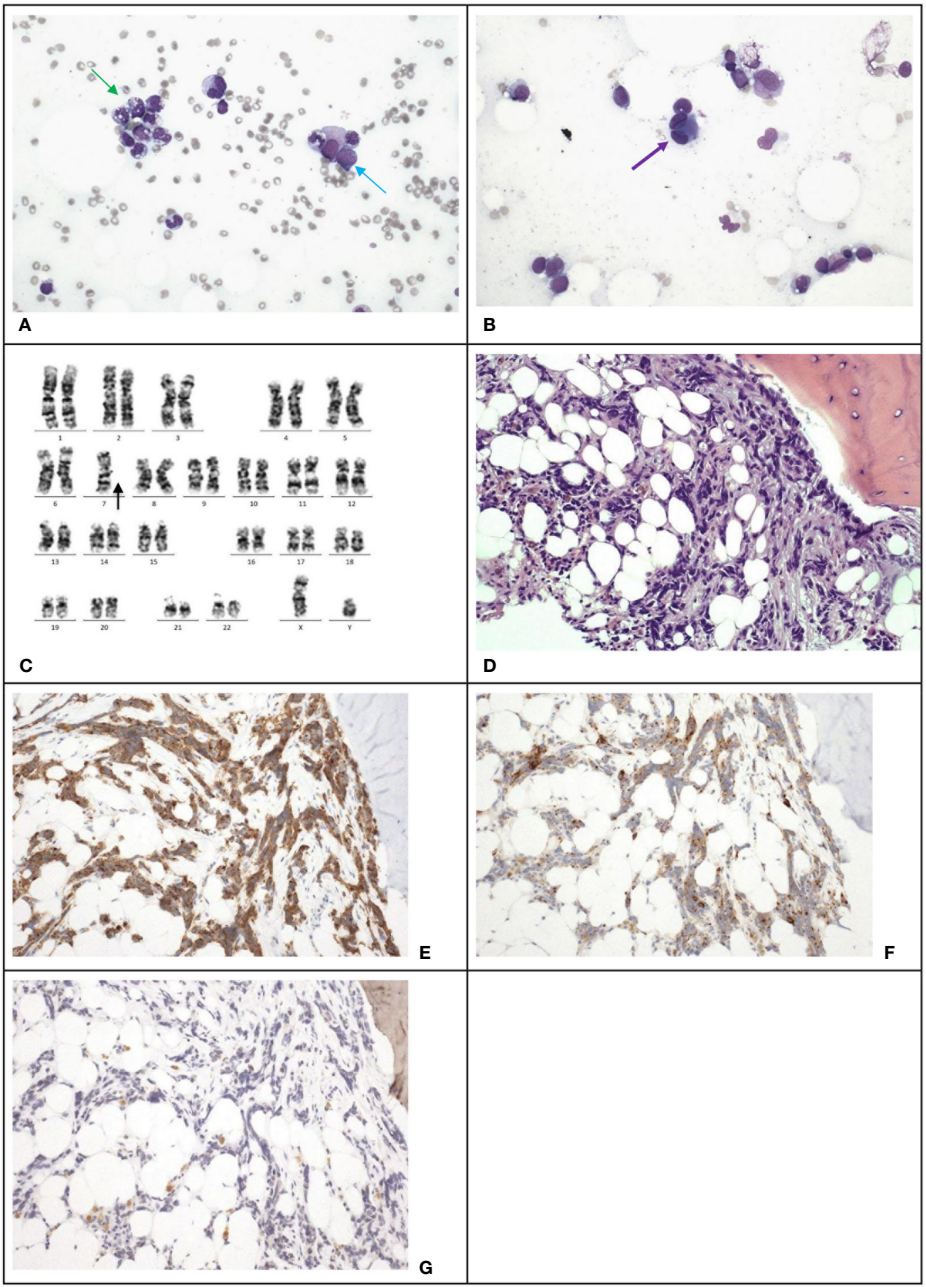
Case 3. **(A)**, The bone marrow demonstrates dysgranulopoiesis (green arrow) and increase in blasts (blue arrow). Wright-Giemsa stain, 400 X magnification. **(B)**, The bone marrow also demonstrates dysmegakaryopoiesis (purple arrow). Wright-Giemsa stain, 400 X magnification. **(C)** Karyotyping reveals a clone with monosomy 7. **(D)**, The trephine biopsy shows non-hemic cellular infiltration. Hematoxylin & Eosin stain, 200 X magnification. Immunohistochemical staining shows the abnormal infiltration is positive for synaptophysin **(E)** and chromogranin **(F)**, and negative for NeuN **(G)**, which is compatible with marrow involvement by medulloblastoma. 200 X magnification.

However, 13 months after the relapse of medulloblastoma, chemotherapy was stopped due to pancytopenia. Marrow examination revealed acute myeloid leukemia (myelodysplasia-related, post cytotoxic therapy), with blasts constituting 23% of all cells through multiparametric flow cytometry, increased myeloblasts comprising >20% of nucleated cells in the trephine biopsy (**Figures 3A, B**), **Table 2** and monosomy 7 by cytogenetic analysis (**Figure 3C**). A single course of azacitidine and venetoclax was administered, but chemotherapy was discontinued due to invasive fungal infection. A repeated marrow examination at 3 months from diagnosis of MDS showed marrow involvement by medulloblastoma (**Figure 3D**) positive for synaptophysin (**Figure 3E**) and chromogranin (**Figure 3F**), and negative for NeuN (**Figure 3G**). The patient was put on palliative care instead of curative approach. The patient’s condition deteriorated with progressive medulloblastoma, and passed away 4 months after the MDS diagnosis, marking 6 years and 9 months since the initial diagnosis of medulloblastoma.

## 3 Discussion

Treatment outcomes for medulloblastoma have improved with the systematic use of chemoradiation, with a 70-80% survival rate for localized disease (7). However, therapy-related myeloid neoplasms (t-MNs) can be a potential complication of treatment for medulloblastoma (8). MDS is a group of disorders characterized by abnormal blood cell production in the bone marrow, usually diagnosed in older adults. Therapy-related MDS (t-MDS) accounts for 10-15% of all MDS cases. The most common subtype of t-MDS is refractory anemia with excess blasts type (RAEB) (9), as was the case in our first patient.

Alkylating agents and topoisomerase II inhibitors are known to contribute to t-MNs. Previous studies have reported cases of t-MDS after treatment for primary brain tumors, and some cases progressed to secondary leukemia (2). In another long-term survival study of patients with recurrent medulloblastoma (10), with a MEMMAT-like approach, 5/29 patients developed secondary hematological malignancies. In general, the median time to development of t-MDS/AML is 3 to 5 years, with the risk decreasing after the first decade (11). Alkylating agents can affect marrow stem cells due to a relative deficiency in the DNA repair protein, O6-Alkylguanine-DNA alkyltransferase, resulting in chromosomal abnormalities with copy number changes. Our three cases received lomustine as standard upfront therapy and they all showed such karyotypic changes. Topoisomerase inhibitors, such as etoposide used in Case 3, generate DNA strand breaks and also chromosomal aberrations and increase the risk of secondary malignancies (12). Of note, topoisomerase inhibitors including etoposide are associated with increased risk of leukemia bearing balanced translocations and inversions which was not seen in case 3. Instead, monosomy 7 was detected in this case (**Figure 3C**) which is typically associated with alkylating agents used in the initial treatment. Radiotherapy may contribute to the development of t-MDS due to exposure of active bone marrow (13). The combination of chemotherapy and whole brain radiation was proposed to increase the risk of t-AML compared to chemotherapy alone (14). Few cases of t-MNs have been solely attributed to radiotherapy (15, 16).

Certain genetic predisposition syndromes also increase the risk of t-MNs (17). In the St. Jude series (18), germline abnormalities were found in 29% of t-MDS/t-AML patients, including LFS, mismatch repair deficiency, neurofibromatosis type 1, Fanconi anemia, and Down syndrome. In a case series, *TP53* point mutations were present in 24% of t-MDS/t-AML cases (19). LFS is typically associated with hypodiploid ALL and AML, while it rarely involves t-MDS, with few reported cases (20). For the clinical characteristics of LFS associated t-MDS, the number of reported cases is small. Talwalkar et al. reported 3 cases of t-MDS with LFS, with latency period from primary cancer ranging from 1.5 years to 4 decades. In general, *TP53* mutations in t-MDS are associated with complex cytogenetic findings, such as those seen in Case 2 (**Figures 2B, C**), and frequently involve chromosome 5 or 7, which was suggested to lead to a poorer prognosis (21). Moreover, *TP53* mutations have been found to confer an inferior overall survival in patients with t-MDS. It was historically suggested that the cytotoxic effect of chemotherapy induced *TP53* mutations, leading to t-MNs (22). However recent studies have proposed that pre-existing progenitors carrying prior *TP53* mutation may be preferentially selected, leading to cytogenetic abnormalities and poor responses to chemotherapy, as seen in patients with t-MNs (23). The linkage between germline *TP53* mutation and latency period to the development of t-MNs remains to be further elucidated. Nevertheless, it has been documented that *TP53* mutations are enriched among medulloblastomas with SHH subgroup and are associated with poorer outcome (24), as shown in cases 1 and 2. Coupled with this, genetic predisposition syndromes such as *TP53* carry a significant prognostic impact and account for treatment failure in these patients.

Case 3 showed a heterozygous germline *MUTYH* variant. The *MUTYH* gene, which encodes for the mutY DNA glycosylase protein, is involved in base excision repair in the DNA repair system. Biallelic inactivating mutations in *MUTYH*, either homozygous or compound heterozygous, are associated with gastrointestinal adenomas and carcinomas, known as the gastrointestinal polyposis syndrome (FAP2) (25). While brain tumors are rare in *MUTYH* mutations (26), Kline et al. (27) identified two pediatric patients with homozygous *MUTYH* mutations, one diagnosed with grade IV medulloblastoma, where loss of heterozygosity (LOH) as a result of chromosome 1p deletion was found. The *MUTYH* variant identified was NM 001048171.1 c.892-2 A>G, identical as that detected in our third patient. The oncogenicity this gene variant in the heterozygous setting warrants further evaluation (28).

In conclusion, we present three cases of early onset t-MN after treatment for medulloblastoma with confirmed or potential contribution by germline predisposition. This highlights the relevance of surveillance for hematological abnormalities in medulloblastoma survivors. While t-MNs are rare, it is important to recognize and monitor in particular for patients with underlying cancer genetic syndromes.

## Data availability statement

The original contributions presented in the study are included in the article/supplementary material. Further inquiries can be directed to the corresponding author.

## Ethics statement

The studies involving humans were approved by Hong Kong Children’s Hospital Research Ethics Committee (HKCH-REC-2020-068). The studies were conducted in accordance with the local legislation and institutional requirements. Written informed consent for participation in this study was provided by the participants’ legal guardians/next of kin. Written informed consent was obtained from the individual(s), and minor(s)’ legal guardian/next of kin, for the publication of any potentially identifiable images or data included in this article.

## Author contributions

LM: Writing – original draft, Writing – review & editing, Conceptualization, Data curation, Formal analysis. VL: Writing – original draft, Writing – review & editing, Data curation, Formal analysis, Investigation, Visualization. WC: Writing – original draft, Writing – review & editing, Data curation. AWKL: Writing – original draft, Writing – review & editing, Data curation. DC: Writing – original draft, Writing – review & editing, Data curation. LY: Writing – original draft, Writing – review & editing, Data curation, Formal analysis, Investigation. JS: Writing – original draft, Writing – review & editing, Data curation, Formal analysis, Investigation. SH: Writing – original draft, Writing – review & editing, Conceptualization. APYL: Funding acquisition, Writing – original draft, Writing – review & editing, Conceptualization, Data curation, Investigation, Supervision.
